# Health impacts of climate change on children and adolescents: A protocol for review of reviews

**DOI:** 10.1371/journal.pone.0352978

**Published:** 2026-07-02

**Authors:** Rilwan Yahaya, Salifu Sharif Alhassan, Rosemary Sitsofe Ayebi-Arthur, Regina Boatemaa Berchie, Edward Wilson Ansah

**Affiliations:** Department of Health, Physical Education and Recreation, University of Cape Coast, Cape Coast, Ghana; La Trobe University - Bundoora Campus: La Trobe University, AUSTRALIA

## Abstract

**Introduction:**

Climate change is a contemporary phenomenon of grave concern to global public health. Climate change events such as droughts, wildfires, tornadoes, heatwaves, floods, sea level rise, hurricanes, tropical cyclones, landslides, extreme rainfall, typhoons, dust storms, and desertification significantly affect local, regional, and global living conditions. In Sub-Saharan Africa, the most disturbing of these are desertification, droughts, and floods, which directly threaten water supplies, food security, and the livelihoods of millions of people. The climate crisis affects the health of older people, adults, children, and adolescents. However, climate-related events are gravely affecting the current and future health and well-being of children and adolescents. Although evidence exists, its integration is vital for policy and practice to protect children and adolescents in the ever-changing climate. Therefore, this review aims to map the existing reviews of the impact of climate change on the health and well-being of children and adolescents.

**Method:**

This review will be conducted according to Arksey and O’Malley’s [36] recommendations and will be reported according to the Preferred Reporting Items for Systematic Reviews and Meta-Analyses Extension for Scoping Reviews (PRISMA-ScR). Scopus, JSTOR, Web of Science, PubMed, Embase and Cochrane Library will be searched to identify relevant records for inclusion in this review. Additional searches will be conducted in Google Scholar and Google for other relevant articles. The review protocol is registered at Open Science Framework: (https://doi.org/10.17605/OSF.IO/A7DEQ).

**Analysis:**

Extracted data will be analysed using thematic content analysis, where data are summarised and qualitatively synthesized according to the recommendations of PRISMA-ScR and Tricco et al. [37]. The results and findings regarding the impacts of climate change on the health and safety of children and adolescents will be compiled, categorized, and presented using a qualitative narrative synthesis.

## Introduction

Climate change has become a major global public health of the twenty-first century, affecting individuals of all age groups, from children to adolescents, to adults, and the elderly [1–3]. The present and future health and well-being of children and adolescents are disproportionally threatened by global climate change crisis [4], because children grow optimally in stable homes, communities, schools, and neighbourhoods [5], settings that are being compromised by the climate crisis.

Climate change refers to a gradual shift in atmospheric conditions, manifesting in extreme weather patterns, including rising temperatures, droughts, wildfires, tornadoes, heatwaves, floods, rising sea levels, hurricanes, tropical cyclones, landslides, extreme rainfall, typhoons, dust storms, desertification, food insecurity, and other phenomena that profoundly and negatively affect local, regional, and global living conditions [6,7].

Research indicates that the climate crisis is predominantly driven by human activities, including the burning of fossil fuels for energy production, industrial operations, rapid urban expansion, and large-scale deforestation, all of which are intensifying harmful changes in the Earth’s atmosphere [6–10]. The consequences of these activities are felt most by the very vulnerable people, such as children and adolescents.

The rate and severity of life-threatening weather events like droughts, storms, floods, heatwaves and wildfires have increased considerably owing to the adverse changing climate further causing community disruptions, property damage, climate-related illnesses, and fatalities, becoming more frequent, and intense [11–13]. For example, The 2023 wildfires in Hawaii and floods in Vermont demonstrated how climate change is intensifying the frequency and severity of extreme weather events, causing devastating impacts on lives, infrastructure, and the natural environment [13–15]. Climate change has already pushed over 600 million people, approximately 9% of the global population, outside the ‘human climatic niche’, the range of temperatures historically conducive to human settlement and wellbeing. Projections indicate that under current warming trajectories of approximately 2.7°C, between one and three billion people could be living outside these habitable climate conditions by the end of this century, facing escalating exposure to extreme heat, food insecurity, and potential displacement. [16,17]. Unfortunately, Children and adolescents are disproportionately vulnerable to the health impacts of climate change, as they are physically and physiologically less equipped than adults to withstand extreme weather events and climate-sensitive diseases such as cholera, malaria, dengue, and Zika. According to WHO estimates, children under five bear 88% of the global climate-related disease burden. [18–20].

Climate change significantly affects the well-being, health, and safety of adolescents and children because of their sensitivity to environmental risks. Damage sustained during these crucial periods may have long-term effects on them [19–22]. Moreover, children are particularly vulnerable to environmental changes because of their smaller stature, physiological and cognitive fragility, and complete reliance on caregivers for safety and protection [21,23]. Children have the right to survival, education, good health, quality nutrition, and welfare, recognized and upheld by the Sustainable Development Goals (SDGs) and the Convention on the Rights of the Child [22–25]. Unfortunately, climate events are threatening and undermining children’s and adolescents’ fundamental human rights. For example, Climate change contributes to increased risk of malaria transmission, childhood undernutrition, diarrheal disease, and adverse mental health outcomes. These climate-sensitive health risks are associated with higher under-five mortality and with rising rates of childhood wasting and stunting in vulnerable regions. [24–27].

Recent reviews demonstrate that climate change affects children’s health through both direct and indirect pathways, thereby increasing morbidity and mortality from climate-sensitive diseases [21,27–29]. Although these findings are significant, the evidence remains scattered and incomplete, making it hard to draw clear conclusions for policy and practice. Therefore, a comprehensive review of existing reviews is needed to better understand how the climate crisis affects the short- and long-term health of children and adolescents, and to guide future policies and interventions. A review of existing reviews is necessary to thoroughly examine the available evidence on how climate change affects the health of children and adolescents, helping to build a clearer and more complete picture to guide future research, policy, and practice. Fortunately, a number of scoping, narrative, and other forms of review have made meaningful contributions to the existing body of knowledge, offering preliminary insights into the impacts of climate change on the health and well-being of children and adolescents [21,27,28]. These reviews suggest that children and adolescents from low-income and vulnerable communities are most severely affected by climate change. In addition to physical health risks, climate change increases their likelihood of developing mental health problems, substance addiction, and suffering unintentional injuries [30–32].

Although research on climate change and child health is expanding, comprehensive, review-based syntheses remain limited [33,34], creating crucial gaps in the evidence. The health effects of climate change on children and adolescents have not been fully explored, especially for those in low- and middle-income countries (LMICs), and indigenous communities, who are often overlooked in existing reviews. While some reviews provide valuable evidence [24,27,28,35], differences in their methodologies hinder comparison and reduce their usefulness for policy and practice. Therefore, a review of reviews is needed to compile the current evidence into a clear, accessible resource for policymakers and practitioners and to systematically identify existing gaps. This will assist in establishing priorities for future research.

Protocols are an important approach to providing a comprehensive method for reviewing existing evidence. Protocols like the current one serve as methodological maps for the main review, which aims to aid future researchers who may want to replicate the study. When protocols are published, they help other researchers with similar ideas avoid duplicating existing reviews and instead identify gaps in the literature. Therefore, there is a need for up-to-date critical evidence to strengthen the case for research, policy, and evidence-based interventions that protect and promote the health, safety, and well-being of children and adolescents amid ever-worsening climate variability. Other researchers, government officials, and policymakers will find this helpful synthesis. The aim of the review is to gather current evidence from existing reviews on the effects of climate change on the well-being, health, and safety of children and adolescents.

### Method and materials

This review of reviews follows the guidelines of Arksey and O’Malley [36]. The review includes the following steps: (1) identifying and stating research objectives; (2) identifying relevant studies; (3) study selection; (4) data collection; (5) data summary and synthesis of results; and (6) consultation. We report the results and findings of this review in accordance with the Preferred Reporting Items for Systematic Reviews and Meta-Analyses Extension for Scoping Reviews (PRISMA-ScR) standards [37]. The review protocol is registered on the Open Science Framework (https://doi.org/10.17605/OSF.IO/A7DEQ).

The Arksey and O’Malley guidelines provide a clear, systematic methodological framework for conducting a scoping study and ensure thorough evaluation by requiring researchers to document their activities and decisions systematically [36]. The guidelines facilitate collaboration among researchers by providing a common framework and terminology for conducting and reporting scoping reviews. Arksey and O’Malley’s framework can be applied across a wide range of subjects, making it an excellent resource for researchers. These principles guide future research and policy decisions while emphasising the need to identify research gaps and areas that require further exploration. The guidelines also promote transparency in the review process, which could increase the validity and trustworthiness of review results [36].

### Research questions

The review will be guided by the following three research questions: (1) What are the impacts of climate change on the health and safety of adolescents and children? (2) What factors make adolescents and children more vulnerable to climate change impacts? (3) What health promotion and public health interventions are available to protect adolescents and children from climate change impacts?

### Search strategy

A comprehensive search will be conducted across six databases: Scopus, PubMed, Cochrane Library, Web of Science, JSTOR, and Embase. The goal is to identify relevant review articles for the current review. An additional search will be conducted in Google Scholar and Google for other relevant articles that could be selected for inclusion in the review. We developed a search strategy that used keywords, Medical Subject Headings (MeSH) terms, and controlled vocabularies for the initial PubMed search (see S1 Table in S1 File). After that, the search terms were modified to allow further database searches (Scopus, Web of Science, JSTOR, Embase, and Cochrane Library). This is necessary because each database has a unique search strategy that requires specific search terms to retrieve highly relevant results.

We intend to extract appropriate peer-reviewed English-language papers published after January 2000 to retrieve up-to-date, relevant articles for the current review. This is because, although climate change issues have been published for several decades, the popularity of health-related climate change literature has increased in recent years. The main study is expected to be completed by the end of June 2026. Details on the timeline can be found in S1 Appendix in S1 File.

### Study selection

The retrieved records will be imported into Mendeley, where duplicate records will be removed. Moreover, a three-stage screening process will be applied to the remaining records to select appropriate published reviews for the study. First, six graduate students will screen the titles and abstracts of the retrieved records to identify full-text records for further screening. This stage of the screening will be supervised by authors RY and RSAA. The second stage will involve authors RBB, SSA, and RSAA searching the reference lists of selected full-text records to identify further pertinent articles for assessment. This is important because full-text articles are assumed to contain a highly relevant list of references that researchers can use to find additional articles for their current study. The third stage will involve independently screening the full-text records by two groups of authors (RY and SAA; RSAA and RBB), according to the eligibility criteria (See S2 Table in S1 File). These processes ensure that only the most relevant published evidence synthesis peer-reviewed articles are included in the current review. Additionally, there will be cross-over screening, where another group cross-checks the screening done by a group. This process helps to provide valid and reliable results and findings for the review. Led by author EWA, researchers will have weekly meetings to discuss the screening process, and any discrepancies will be resolved by consensus. See the PRISMA flow diagram that represents the selection process (See S1 Fig in S1 File).

### Data extraction and charting

As in primary research, data extraction is a key component of conducting a systematic review. However, while a researcher may collect data in primary research, data extraction for a review requires multiple individuals. There is also a need to prepare, review, and pilot-test the data extraction sheet (See S2 Appendix in S1 File) in line with the study objective or research questions. The aims are to increase consistency in data extraction and improve study validity. It is important to note that the data extraction sheet is not fixed; it evolves as new variables arise while reading the selected articles.

Two authors (RY and RAT) will independently extract data, which will be reviewed by authors SSA and EWA to ensure data accuracy, reliability, and consistency. Author RY will extract data on authors, publication year, study objectives, and the type of review (systematic review, scoping review, rapid review, living systematic review, umbrella review, topical review, and others). Author RBB will gather data on climate change events (extreme heat, floods, droughts, etc.) and the effects of climate change on the health, safety, and well-being of adolescents and children from the selected review articles. Moreover, author RSAA will extract data on reported vulnerability factors and on health promotion or public health initiatives intended to protect adolescents and children from the negative impacts of climate change on their well-being and health. Authors will cross-validate the extractions performed by other authors, and author EWA will review the extracted data to finalise the dataset. The purpose of the review and cross-validation is to improve the consistency and reliability of the extracted data. This also helps ensure that the extracted data are complete and accurate and represent our stated research questions.

### Quality assessment

Methodological quality assessment is important for the review articles that are selected and included in synthesised studies. Thus, we will use AMSTAR – a measurement tool to assess the methodological quality of the selected and included reviews [38]. AMSTAR is an 11-item validated quality appraisal instrument [39] for assessing published review papers. The 11-items for assessing review studies included; whether review questions and inclusion criteria established before the review; duplicate study selection and data extraction performed; comprehensive literature search performed; publication (i.e., grey literature) used as an inclusion criterion; list of studies (included and excluded) provided; characteristics of the included studies provided; scientific quality of the included studies assessed and documented; scientific quality of the included studies used appropriately in formulating conclusions; methods used to combine the findings of studies appropriate; likelihood of publication bias assessed; and whether conflict of interest stated [39]. Each of the 11-item assessment criteria is used to rate published review articles as Yes, No, I don’t know, or Not Applicable [39], which we applied to assess the quality of the included review articles.

To ensure reporting transparency and strict adherence to protocols, a finalised PRISMA-P 2015 checklist is provided for this scoping review of reviews examining the health impact of climate change on children and adolescents (See S3 Appendix).

### Data analysis and synthesis

Extracted data will be analysed using thematic content analysis, where data are summarised and qualitatively synthesised according to the recommendations of Arksey and O’Malley [36] and PRISMA-ScR [37]. As a result, we will summarise, categorise, and present the results and findings on the interactions between climate change events and the health, safety, and well-being of children and adolescents. This is a methodical procedure in which the data is coded and categorised into themes and sub-themes in accordance with the stated questions [40,41]. The initially provided descriptive codes serve as the basis for creating more detailed codes to answer the stated research questions [41]. This approach aims to enhance understanding of how climate change events affect the health and well-being of children and adolescents, as reported in published review papers.

The findings from the analysis then allow us to synthesise, present, and provide an in-depth discussion to draw conclusions and recommendations concerning the effects of climate change on the health and well-being of children and adolescents, as well as the policy and practice relevance of the findings. Using a qualitative synthesis enables the researcher to explore the short- and long-term effects of climate distress on the health and well-being of children and adolescents. Besides, this qualitative synthesis and narrative analysis help distil the gap in the research literature and highlight the needed directions for future research, as discussed in our review of the findings.

### Ethical approval and informed consent

Ethical approval is not required because no primary data will be collected and no patient involvement will occur. We expect to publish the findings of this article in a reputable journal and/or present them at climate change conferences and workshops. The protocol is registered with OSF (https://doi.org/10.17605/OSF.IO/A7DEQ).

### Consultation

Consultations are important for carrying out scoping reviews. We consulted Dr Mustapha Amoadu, a Research Fellow, who also reviewed both the search teams and the search strategies and may review the extracted data. He is a review expert and a researcher in climate change and health. Additionally, we are consulting a chartered librarian at the Sam Jonah Library of the University of Cape Coast, who is helping to design the search and to locate relevant records.

## Supporting information

S1 File S1 Table. This legend explains the search strategies used across six databases (PubMed, Web of Science, Scopus, JSTOR, Cochrane Library, Embase) for a scoping review on climate change impacts on children and adolescents. Core components: #1 = Climate change terminology (global warming, floods, droughts, air pollution, etc.) #2 = Health outcomes (disease, mental health, respiratory, cardiovascular, malnutrition, etc.) #3 = Pediatric populations (children, adolescents, youth, infants, schoolchildren). #4 = Review study types (systematic reviews, scoping reviews, meta-analyses, evidence syntheses). Combined logic: (#1 AND #2 AND #4 NOT animal) OR (#1 AND #3 AND #4 NOT animal). Technical elements explained: Field descriptors – How to search specific record sections (e.g., [tiab] = title/abstract, TS = topic search, [MeSH] = controlled vocabulary). Boolean operators – AND (narrow), OR (broaden), NOT (exclude). Wildcards – * for multiple characters (e.g., flood* catches flooding, floods, flooded). Filters: English language only, publications from 2000 onwards. S2 Table. This table presents the eligibility criteria for a scoping review on climate change’s health impacts on children and adolescents. Inclusion: Peer-reviewed reviews (scoping, systematic, rapid, etc.) published in English from 2000–2025 examining how climate change physically and mentally affects ages 0–19. Exclusion: Studies focused only on adults, grey literature, primary research, non-English publications, and incompatible review types (meta-analyses, reviews of reviews, etc.). S1 Fig. PRISMA flow diagram of the study selection process for identifying relevant review articles on the health impacts of climate change on children and adolescents. Records were identified by searching six electronic databases (Scopus, PubMed, Cochrane Library, Web of Science, JSTOR, and Embase), supplemented by searches of Google Scholar and Google. Following deduplication in Mendeley reference management software, records underwent three sequential screening stages: (1) title and abstract screening by six graduate students supervised by authors RY and RSAA; (2) reference list searching of eligible full-text records conducted by authors RBB, SSA, and RSAA; and (3) independent full-text screening by two author groups (RY and SAA; RSAA and RBB) against the pre-defined eligibility criteria. Cross-over screening and consensus-based resolution of discrepancies, led by author EWA, were applied throughout. PRISMA, Preferred Reporting Items for Systematic Reviews and Meta-Analyses. S1 Appendix. Timelines for the review process. This table presents the planned schedule for each major phase of the scoping review of reviews on the health impacts of climate change on children and adolescents, from literature searching and proposal registration through to manuscript finalisation and journal submission, covering the period January to June 2026. S2 Appendix. Scoping Review Data Extraction Sheets. Sheet 1: Captures bibliographic details (authors, year, title) and methodological scope (databases, search dates, number of studies, climate exposures, research questions). Sheet 2: Extracts health outcomes (mental and physical), vulnerability factors, interventions/initiatives, evidence gaps, policy/research recommendations, and authors’.
(ZIP)

S3 Appendix A completed PRISMA-P 2015 checklist detailing protocol adherence and transparent reporting standards for this scoping review of reviews. The checklist includes 17 items covering protocol registration, review rationale, objectives, eligibility criteria, information sources, data extraction methodology, and planned analyses, ensuring comprehensive and reproducible review conduct.
(DOCX)
