# Supplementary material for: Health impacts of climate change on children and adolescents: A protocol for review of reviews
Source: PLoS One. 2026 Jul 2;21(7):e0352978. doi: 10.1371/journal.pone.0352978 (PMC13327113; doi:10.1371/journal.pone.0352978)
Supplement: S1 File — This legend explains the search strategies used across six databases (PubMed, Web of Science, Scopus, JSTOR, Cochrane Library, Embase) for a scoping review on climate change impacts on children and adolescents. Core components: #1 = Climate change terminology (global warming, floods, droughts, air pollution, etc.) #2 = Health outcomes (disease, mental health, respiratory, cardiovascular, malnutrition, etc.) #3 = Pediatric populations (children, adolescents, youth, infants, schoolchildren). #4 = Review study types (systematic reviews, scoping reviews, meta-analyses, evidence syntheses). Combined logic: (#1 AND #2 AND #4 NOT animal) OR (#1 AND #3 AND #4 NOT animal). Technical elements explained: Field descriptors – How to search specific record sections (e.g., [tiab] = title/abstract, TS = topic search, [MeSH] = controlled vocabulary). Boolean operators – AND (narrow), OR (broaden), NOT (exclude). Wildcards – * for multiple characters (e.g., flood* catches flooding, floods, flooded). Filters: English language only, publications from 2000 onwards. S2 Table. This table presents the eligibility criteria for a scoping review on climate change’s health impacts on children and adolescents. Inclusion: Peer-reviewed reviews (scoping, systematic, rapid, etc.) published in English from 2000–2025 examining how climate change physically and mentally affects ages 0–19. Exclusion: Studies focused only on adults, grey literature, primary research, non-English publications, and incompatible review types (meta-analyses, reviews of reviews, etc.). S1 Fig. PRISMA flow diagram of the study selection process for identifying relevant review articles on the health impacts of climate change on children and adolescents. Records were identified by searching six electronic databases (Scopus, PubMed, Cochrane Library, Web of Science, JSTOR, and Embase), supplemented by searches of Google Scholar and Google. Following deduplication in Mendeley reference management software, records underwent thre [file pone.0352978.s001.zip › S1 Table.docx]

**S1 Table: Search Strategies**

|  | **PubMed** - **Search Strategy** |
| --- | --- |
| #1 Search to find climate change | ("Climate Change"[MeSH] OR "climate change"[tiab] OR "global warming"[tiab] OR "climate variability"[tiab] OR "extreme weather"[tiab] OR "extreme heat"[tiab] OR "heat wave*"[tiab] OR "heatwave*"[tiab] OR "flood*"[tiab] OR "drought*"[tiab] OR "wildfire*"[tiab] OR "bushfire*"[tiab] OR "air pollution"[tiab] OR "greenhouse gas*"[tiab] OR "sea level rise"[tiab] OR "climate crisis"[tiab] OR "climate emergency"[tiab] OR "rising temperature*"[tiab] OR "environmental change*"[tiab] *" OR planetary health*"[tiab] OR environmental sustainability*"[tiab] ) |
| # 2 Search to find Health impact | ("Health"[MeSH] OR "Public Health"[MeSH] OR "Mental Health"[MeSH] OR "Communicable  Diseases"[MeSH] OR "Respiratory Tract Diseases"[MeSH] OR "Cardiovascular Diseases"[MeSH] OR "Malnutrition"[MeSH] OR "health impact*"[tiab] OR "health effect*"[tiab] OR "health outcome*"[tiab] OR "health risk*"[tiab] OR "disease burden"[tiab] OR "morbidity"[tiab] OR "mortality"[tiab] OR "mental health"[tiab] OR "wellbeing"[tiab] OR "well-being"[tiab] OR "respiratory"[tiab] OR "cardiovascular"[tiab] OR "vector-borne"[tiab] OR "waterborne"[tiab] OR "heat-related illness*"[tiab] OR "heat stress"[tiab] OR "malnutrition"[tiab] OR "food insecurity"[tiab] OR "asthma"[tiab] OR "allerg*"[tiab] OR "diarrhea*"[tiab] OR "diarrhoea*"[tiab] OR "infectious disease*"[tiab] OR "anxiety"[tiab] OR "depression"[tiab] OR "psychological"[tiab] OR "injury"[tiab]) |
| #3 Search to find children and Adolescents | ("Child"[MeSH] OR "Adolescent"[MeSH] OR "Infant"[MeSH] OR "Child, Preschool"[MeSH] OR "Pediatrics"[MeSH] OR "child*"[tiab] OR "adolescent*"[tiab] OR "youth"[tiab] OR "young people"[tiab] OR "pediatric*"[tiab] OR "paediatric*"[tiab] OR "infant*"[tiab] OR "newborn*"[tiab] OR "neonate*"[tiab] OR "teenager*"[tiab] OR "school-age"[tiab] OR "schoolchildren"[tiab] OR "under-5"[tiab] OR "minors"[tiab]) |
| #4 Search to identify review studies | ("Systematic Reviews as Topic"[MeSH] OR "Meta-Analysis as Topic"[MeSH] OR "systematic review*"[tiab] OR "scoping review*"[tiab] OR "meta-analysis"[tiab] OR "meta-analysis"[tiab] OR "umbrella review*"[tiab] OR "review of reviews"[tiab] OR "overview of reviews"[tiab] OR "narrative review*"[tiab] OR "integrative review*"[tiab] OR "rapid review*"[tiab] OR "evidence synthesis"[tiab] OR"literature review*"[tiab] OR systematic review[pt] OR meta-analysis[pt]) |
| Broader search strategy | #1 AND #2 AND #4 AND NOT animal*  #1 AND #3 AND #4 AND NOT animal*  (Filters activated: English, from 2000/01/01) |
|  | **Web of Science -** **Search Strategy** |
| # 1 Search to find climate change | TS=("climate change" OR "global warming" OR "climate variability" OR  "climate crisis" OR "climate emergency" OR "extreme weather" OR  "extreme heat" OR "heat wave*" OR "heatwave*" OR "flood*" OR  "drought*" OR "wildfire*" OR "bushfire*" OR "air pollution" OR  "rising temperature*" OR "temperature increase*" OR "sea level rise" OR  "greenhouse gas*" OR "environmental change*") |
| # 2 Search to find Health impact | TS=("health impact*" OR "health effect*" OR "health outcome*" OR  "health risk*" OR "disease burden" OR "morbidity" OR "mortality" OR  "mental health" OR "wellbeing" OR "well-being" OR "respiratory" OR  "cardiovascular" OR "vector-borne" OR "waterborne" OR "heat stress" OR  "heat-related illness*" OR "malnutrition" OR "food insecurity" OR  "undernutrition" OR "asthma" OR "allerg*" OR "diarrhoea*" OR  "diarrhoea*" OR "infectious disease*" OR "anxiety" OR "depression" OR  "psychological" OR "injury" OR "displacement" OR "communicable disease*") |
| # 3 Search to identify Children and Adolescents | TS=("child*" OR "adolescent*" OR "youth" OR "young people" OR  "young person*" OR "pediatric*" OR "paediatric*" OR "infant*" OR  "newborn*" OR "neonate*" OR "teenager*" OR "school-age" OR  "schoolchildren" OR "under-5" OR "under five" OR "minors") |
| # 4 Search to identify Review studies | TS=("systematic review*" OR "scoping review*" OR "meta-analysis" OR  "meta-analysis" OR "umbrella review*" OR "review of reviews" OR  "overview of reviews" OR "narrative review*" OR "integrative review*" OR  "rapid review*" OR "evidence synthesis" OR "literature review*") |
| Broader search strategy | #1 AND #2 AND #3 and not animal" #1 AND #3 AND #4 and not animal" (English filters activated, 2000/01/01) |
|  | **Scopus - Search Strategy** |
| # 1 Search to identify climate change | ("climate change" OR "global warming" OR  "climate variability" OR "climate crisis" OR  "climate emergency" OR "extreme weather" OR  "extreme heat" OR "heat wave*" OR "heatwave*" OR  "flood*" OR "drought*" OR "wildfire*" OR  "bushfire*" OR "air pollution" OR  "rising temperature*" OR "temperature increase*" OR  "sea level rise" OR "greenhouse gas*" OR  "environmental change*" OR "climate hazard*" OR  "climate-related" OR "climate impact*" OR  "climate threat*" OR "climate risk*") |
| # 2 Search to identify Health impact | ("health impact*" OR "health effect*" OR "health outcome*" OR "health risk*" OR "disease burden" OR "morbidity" OR "mortality" OR "mental health" OR "wellbeing" OR "well-being" OR "respiratory" OR "cardiovascular" OR "vector-borne" OR "waterborne" OR "heat stress" OR "heat-related illness*" OR "heat-related death*" OR "malnutrition" OR "food insecurity" OR "undernutrition" OR "asthma" OR "allerg*" OR "diarrhea*" OR "diarrhoea*" OR "infectious disease*" OR "communicable disease*" OR "anxiety" OR "depression" OR "psychological" OR "psychosocial" OR "injury" OR "displacement" OR "trauma" OR "eco-anxiety" OR "climate anxiety" OR "stunting" OR "wasting" OR "malaria" OR "dengue" OR "cholera" OR "skin disease*" OR "eye disease*" OR "premature death*" OR  "hospital admission*" OR "emergency visit*") |
| # 3 Search to identify Children and Adolescents | ("child*" OR "adolescent*" OR  "youth" OR "young people" OR "young person*" OR  "pediatric*" OR "paediatric*" OR "infant*" OR  "newborn*" OR "neonate*" OR "neonatal" OR  "teenager*" OR "school-age" OR "schoolchildren" OR  "under-5" OR "under five" OR "minors" OR  "toddler*" OR "preschool*" OR "pre-school*" OR  "school children" OR "juvenile*" OR  "early childhood" OR "perinatal" OR  "childhood" OR "adolescence") |
| # 4 Search to identify Review studies | ("systematic review*" OR "scoping review*" OR "meta-analysis" OR "meta-analysis" OR "umbrella review*" OR "review of reviews" OR "overview of reviews" OR "narrative review*" OR "integrative review*" OR "rapid review*" OR "evidence synthesis" OR "literature review*" OR "systematic map*" OR "evidence map*" OR "realist review*" OR  "mixed method* review*") |
| Overall search strategy | #1 AND #2 AND #3 and not animal" #1 AND #4 AND #4 and not animal"  (English filters activated, 2000/01/01) |
|  | **JSTOR - Search Strategy** |
| # 1 Search to identify climate change | ("climate change" OR "global warming" OR "climate variability" OR  "climate crisis" OR "climate emergency" OR "extreme weather" OR  "extreme heat" OR "heatwave*" OR "heat wave*" OR "flood*" OR  "drought*" OR "wildfire*" OR "bushfire*" OR "air pollution" OR  "greenhouse gas*" OR "sea level rise" OR "environmental change*" OR  "climate impact*" OR "climate hazard*") |
| # 2 Search to identify health impact | ("health impact*" OR "health effect*" OR "health outcome*" OR  "disease burden" OR "mental health" OR "respiratory" OR  "cardiovascular" OR "vector-borne" OR "malnutrition" OR  "food insecurity" OR "infectious disease*" OR "communicable disease*" OR  "heat-related illness*" OR "anxiety" OR "depression" OR  "psychological" OR "psychosocial" OR "eco-anxiety" OR  "climate anxiety" OR "morbidity" OR "mortality") |
| # 3 Search to identify children and Adolescents | ti:("child*" OR "adolescent*" OR "youth" OR "young people" OR  "pediatric*" OR "paediatric*" OR "infant*" OR "newborn*" OR  "neonate*" OR "teenager*" OR "school-age" OR "schoolchildren" OR  "under-5" OR "early childhood" OR "childhood" OR "adolescence" OR  "toddler*" OR "preschool*" OR "minors") |
| # 4 Search to identify review studies | ("systematic review" OR "scoping review" OR "meta-analysis" OR  "umbrella review" OR "review of reviews" OR "overview of reviews" OR  "narrative review" OR "integrative review" OR "rapid review" OR  "evidence synthesis" OR "literature review") |
| Overall search strategy | As stated in the English filters enabled on January 1, 2000, "#1 AND #2 AND #3 and not animal" #1 AND #3 AND #4 and not animal” |
|  | **Cohcrane Library - Search Strategy** |
| # 1 Search to identify climate change | [MeSH descriptor: Climate Change explode all trees] OR  [MeSH descriptor: Global Warming kills all trees] OR  [MeSH descriptor: Air Pollution explode all trees] OR  [MeSH descriptor: Floods explode all trees] OR  [MeSH descriptor: Droughts affect all trees] OR  ("climate change" OR "global warming" OR "climate variability"):ti,ab,kw OR  ("climate crisis" OR "climate emergency" OR "extreme weather"):ti,ab,kw OR  ("extreme heat" OR "heat wave*" OR "heatwave*"):ti,ab,kw OR  ("flood*" OR "drought*" OR "wildfire*" OR "bushfire*"):ti,ab,kw OR  ("air pollution" OR "greenhouse gas*" OR "sea level rise"):ti,ab,kw OR  ("rising temperature*" OR "temperature increase*"):ti,ab,kw OR  ("environmental change*"): ti, ab,kw |
| # 2 Search to identify Health impact | [MeSH descriptor: Child Health explode all trees] OR  [MeSH descriptor: Public Health explode all trees] OR  [MeSH descriptor: Mental Health explode all trees] OR  [MeSH descriptor: Respiratory Tract Diseases explode all trees] OR  [MeSH descriptor: Cardiovascular Diseases explode all trees] OR  [MeSH descriptor: Malnutrition explode all trees] OR  [MeSH descriptor: Communicable Diseases explode all trees] OR  ("health impact*" OR "health effect*" OR "health outcome*"):ti,ab,kw OR  ("health risk*" OR "disease burden" OR "morbidity" OR "mortality"):ti,ab,kw OR  ("mental health" OR "wellbeing" OR "well-being"):ti,ab,kw OR  ("respiratory" OR "cardiovascular" OR "vector-borne"):ti,ab,kw OR  ("waterborne" OR "heat stress" OR "heat-related illness*"):ti,ab,kw OR  ("malnutrition" OR "food insecurity" OR "undernutrition"):ti,ab,kw OR  ("asthma" OR "allerg*" OR "diarrhea*" OR "diarrhoea*"):ti,ab,kw OR  ("infectious disease*" OR "anxiety" OR "depression"):ti,ab,kw OR  ("psychological" OR "injury" OR "displacement"): ti, ab,kw |
| # 3 Search to identify Children and Adolescents | [MeSH descriptor: Child explode all trees] OR  [MeSH descriptor: Adolescent explode all trees] OR  [MeSH descriptor: Infant explode all trees] OR  [MeSH descriptor: Paediatrics explode all trees] OR  ("child*" OR "adolescent*" OR "youth" OR "young people"):ti,ab,kw OR  ("young person*" OR "pediatric*" OR "paediatric*"):ti,ab,kw OR  ("infant*" OR "newborn*" OR "neonate*" OR "teenager*"):ti,ab,kw OR  ("school-age" OR "schoolchildren" OR "under-5"):ti,ab,kw OR  ("under five" OR "minors"): ti, ab,kw |
| # 4 Search to identify Review Studies | [MeSH descriptor: Systematic Reviews as Topic explode all trees] OR  [MeSH descriptor: Meta-Analysis as Topic explode all trees] OR  ("systematic review*" OR "scoping review*"):ti,ab,kw OR  ("meta-analysis" OR "meta-analysis"):ti,ab,kw OR  ("umbrella review*" OR "review of reviews"):ti,ab,kw OR  ("overview of reviews" OR "narrative review*"):ti,ab,kw OR  ("integrative review*" OR "rapid review*"):ti,ab,kw OR  ("evidence synthesis" OR "literature review*"): ti, ab,kw |
| Overall search strategy | #1 AND #2 AND #3 and not animal" #1 AND #3 AND #4 and not animal" (English filters activated, 2000/01/01) |
|  | **Embase - Search Strategy** |
| # 1 Search to identify climate change | exp climate change/ OR  exp global warming/ OR  exp air pollution/ OR  exp flood/ OR  exp drought/ OR  exp wildfire/ OR  'climate change':ti, ab,kw OR  'global warming':ti, ab,kw OR  'climate variability':ti, ab,kw OR  'climate crisis':ti, ab,kw OR  'climate emergency':ti, ab,kw OR  'extreme weather':ti, ab,kw OR  'extreme heat':ti,ab,kw OR  'heat wave*':ti,ab,kw OR  'heatwave*':ti, ab,kw OR  'flood*':ti,ab,kw OR  'drought*':ti, ab,kw OR  'wildfire*':ti, ab,kw OR  'bushfire*':ti, ab,kw OR  'air pollution':ti, ab,kw OR  'rising temperature*':ti, ab,kw OR  'temperature increase*':ti, ab,kw OR  'sea level rise':ti,ab,kw OR  'greenhouse gas*':ti, ab,kw OR  'environmental change*':ti,ab,kw |
| # 2 Search to identify Health impact | exp child health/ OR  exp public health/ OR  exp mental health/ OR  exp respiratory disease/ OR  exp cardiovascular disease/ OR  exp malnutrition/ OR  exp communicable disease/ OR  exp vector-borne disease/ OR  exp heat stroke/ OR  exp food insecurity/ OR  'health impact*':ti, ab,kw OR  'health effect*':ti, ab,kw OR  'health outcome*':ti, ab,kw OR  'health risk*':ti, ab,kw OR  'disease burden':ti, ab,kw OR  'morbidity':ti,ab,kw OR  'mortality':ti,ab,kw OR  'mental health':ti, ab,kw OR  'wellbeing':ti, ab,kw OR  'well-being ': ti, ab,kw OR  'respiratory':ti,ab,kw OR  'cardiovascular ': ti, ab,kw OR  'vector-borne ': ti, ab,kw OR  'waterborne':ti,ab,kw OR  'heat-related illness*':ti, ab,kw OR  'heat stress':ti,ab,kw OR  'malnutrition':ti, ab,kw OR  'food insecurity':ti, ab,kw OR  'undernutrition':ti, ab,kw OR  'asthma':ti, ab,kw OR  'allerg*':ti,ab,kw OR  'diarrhea*':ti,ab,kw OR  'diarrhoea*':ti,ab,kw OR  'infectious disease*':ti, ab,kw OR  'anxiety':ti,ab,kw OR  'depression':ti,ab,kw OR  'psychological ': ti, ab,kw OR  'injury':ti, ab,kw OR  'displacement':ti,ab,kw |
| # 3 Search to identify Children and Adolescents | exp child/ OR  exp adolescent/ OR  exp infant/ OR  exp paediatrics/ OR  exp newborn/ OR  'child*':ti,ab,kw OR  'adolescent*':ti,ab,kw OR  'youth':ti,ab,kw OR  'young people':ti, ab,kw OR  'young person*':ti, ab,kw OR  'pediatric*':ti,ab,kw OR  'paediatric*':ti,ab,kw OR  'infant*':ti,ab,kw OR  'newborn*':ti,ab,kw OR  'neonate*':ti,ab,kw OR  'teenager*':ti,ab,kw OR  'school-age':ti, ab,kw OR  'schoolchildren':ti, ab,kw OR  'under-5':ti, ab,kw OR  'under five':ti, ab,kw OR  'minors':ti,ab,kw |
| # 4 Search to identify Review studies | exp systematic review/ OR  exp meta-analysis/ OR  exp evidence synthesis/ OR  'systematic review'/it OR  'meta-analysis'/it OR  'systematic review*':ti, ab,kw OR  'scoping review*':ti, ab,kw OR  'meta-analysis':ti,ab,kw OR  'meta-analysis':ti,ab,kw OR  'umbrella review*':ti, ab,kw OR  'review of reviews':ti,ab,kw OR  'overview of reviews':ti,ab,kw OR  'narrative review*':ti, ab,kw OR  'integrative review*':ti, ab,kw OR  'rapid review*':ti, ab,kw OR  'evidence synthesis':ti, ab,kw OR  'literature review*':ti,ab,kw |
| Overall search strategy | #1 AND #2 AND #3 and not animal’ #1 AND #3 AND #4 and not animal’ (The English filters were activated from January 1, 2000) |
