# Supplementary material for: Health impacts of climate change on children and adolescents: A protocol for review of reviews
Source: PLoS One. 2026 Jul 2;21(7):e0352978. doi: 10.1371/journal.pone.0352978 (PMC13327113; doi:10.1371/journal.pone.0352978)
Supplement: S1 File — This legend explains the search strategies used across six databases (PubMed, Web of Science, Scopus, JSTOR, Cochrane Library, Embase) for a scoping review on climate change impacts on children and adolescents. Core components: #1 = Climate change terminology (global warming, floods, droughts, air pollution, etc.) #2 = Health outcomes (disease, mental health, respiratory, cardiovascular, malnutrition, etc.) #3 = Pediatric populations (children, adolescents, youth, infants, schoolchildren). #4 = Review study types (systematic reviews, scoping reviews, meta-analyses, evidence syntheses). Combined logic: (#1 AND #2 AND #4 NOT animal) OR (#1 AND #3 AND #4 NOT animal). Technical elements explained: Field descriptors – How to search specific record sections (e.g., [tiab] = title/abstract, TS = topic search, [MeSH] = controlled vocabulary). Boolean operators – AND (narrow), OR (broaden), NOT (exclude). Wildcards – * for multiple characters (e.g., flood* catches flooding, floods, flooded). Filters: English language only, publications from 2000 onwards. S2 Table. This table presents the eligibility criteria for a scoping review on climate change’s health impacts on children and adolescents. Inclusion: Peer-reviewed reviews (scoping, systematic, rapid, etc.) published in English from 2000–2025 examining how climate change physically and mentally affects ages 0–19. Exclusion: Studies focused only on adults, grey literature, primary research, non-English publications, and incompatible review types (meta-analyses, reviews of reviews, etc.). S1 Fig. PRISMA flow diagram of the study selection process for identifying relevant review articles on the health impacts of climate change on children and adolescents. Records were identified by searching six electronic databases (Scopus, PubMed, Cochrane Library, Web of Science, JSTOR, and Embase), supplemented by searches of Google Scholar and Google. Following deduplication in Mendeley reference management software, records underwent thre [file pone.0352978.s001.zip › S2 Appendix.docx]

**Health Impacts of Climate Change on Children and Adolescents: A Review of Reviews**

Extractor's Name………………………………………….

Date of Extraction...........................................................

Extraction Sheet 1

| **Authors / Year/Type of publication** | **Title of review** | **Research Question/Objectives/ Population** | **Databases Searched/ Search Date Range** | **Number Studies/Reviews included** | **Climate Change Exposure(s)** |
| --- | --- | --- | --- | --- | --- |
|  |  |  |  |  |  |
|  |  |  |  |  |  |
|  |  |  |  |  |  |
|  |  |  |  |  |  |
|  |  |  |  |  |  |
|  |  |  |  |  |  |
|  |  |  |  |  |  |

Extraction Sheet 2

| **Mental Health: PTSD/Anxiety/Depression/Eco-anxiety/Climate Distress/General Well-being & Coping** | **Physical Health: Mortality/Morbidity/Respiratory/Infectious Disease/Nutritional & Developmental** | **Key Reported Gaps in Evidence** | **Key Recommendations for Policy/ Research** | **Authors' Overall Conclusion** |
| --- | --- | --- | --- | --- |
|  |  |  |  |  |
|  |  |  |  |  |
|  |  |  |  |  |
|  |  |  |  |  |
|  |  |  |  |  |
|  |  |  |  |  |
