# Supplementary material for: Health impacts of climate change on children and adolescents: A protocol for review of reviews
Source: PLoS One. 2026 Jul 2;21(7):e0352978. doi: 10.1371/journal.pone.0352978 (PMC13327113; doi:10.1371/journal.pone.0352978)
Supplement: Attachment — Submitted filename: Response to Reviewers.docx [file pone.0352978.s004.docx]

**Response to Editor and Reviewers**

**Manuscript title: Health Impacts of Climate Change on Children and Adolescents: A Protocol for Review of Reviews**

**Journal:** PLOS ONE

**Manuscript ID:** PONE-D-25-69159

**Date:** 30^th^ May 2026

Dear Katrina Li,

Thank you for the opportunity to revise and resubmit our manuscript. We are grateful to the Editor and Reviewers for their thorough and constructive comments. The feedback has significantly strengthened the manuscript's rigor, clarity, and comprehensiveness.

Below, we provide a point-by-point response to each comment. All changes to the manuscript are indicated in the revised version. For ease of reference, each comment is reproduced in full, followed by our response and description of the corresponding revision.

**REVIEWER #1: Comments and Responses**

This will make a valuable contribution to the understanding of climate change and children's health. I am unclear if publication of the protocol is necessary or valuable prior to the review being completed.

1. *The language is good but needs substantial revisions prior to publication. I am unable to name all the recommended changes due to number. The abstract and pages 8-10 need to be checked closely for grammar and fluency. I have listed below some specific comments, however these are not exhaustive.*

**Response 1**

Issues with grammar and fluency in the Abstract and on pages 8-10 have been addressed.

2. *Abstract-"Climate change events such as extreme heat, rising sea levels, floods, food insecurity, and others significantly affect local, regional, and global living conditions." These are not all "events"*
   **Response 2**

We have addressed this comment.

3. *"Therefore, this review aims to re-map the existing reviews of the impact of climate change on the health and well-being of children and adolescents." Have the reviews already been mapped? If not then this is a first "mapping" of all reviews rather than "re-map".
   "Climate change refers to a gradual atmospheric shift"- I have not heard this description before. It may be accurate but I'm not certain.*
   **Response 3**

We addressed the sentence on “remap” to reflect our intention of synthesizing existing revies.

Yes, our description of climate change is correct, but we provided further explanation for clarity.

4. *References 5 and 6 likely should be national/international expert reports such as from IPCC/US National Climate Assessment rather than articles focused on single issue (agriculture) or by single author.*

**Response 4**

References 5 and 6 have been improved to include expert reports.

5. *"Evidence suggests that the climate crisis is largely anthropogenic"- this should be more definitively stated. Per IPCC AR6 "Human activities, principally through emissions of greenhouse gases, have unequivocally
   caused global warming"*
   **Response 5**

This statement has been improved to address the concern raised.

6. *"The rate and severity of life-threatening weather events..." the reference is only re extreme rainfall and drought, not the other listed weather events. Each of these events is different, and not all are increasing in both frequency and severity. References should support the statement for each listed event.*
   **Response 6**

More references have been added to support this statement.

7. *"the 2023 wildfires in Hawaii and the floods in Vermont were intense and frequent"-more frequent does not make sense here. It would be better to reference the article referenced by the book for this statement rather than the whole book.*
   **Response 7**

The statement has been improved and an additional reference added.

8. *Please confirm the accuracy of this statement. 3 billion is a high percentage of the world population. "Accordingly, heatwaves will displace up to three billion people by the end of this century [14]."*
   **Response 8**

The statements have been rephrased to clarify their accuracy.

9. *Page 9:"For example, the recent rise in malaria, malnutrition, diarrhea, mental health challenges, and the risk of under-five mortality, as well as increases in wasting and stunting, are associated with climate change [21,23,24]." Are all of these increasing? I do not see that these references support the broad statement.
   7. Page 9 is confusing for a variety of reasons.
   - "implications for higher morbidity and mortality from various climate-related diseases"; "diseases" is not the correct term here, could be "conditions" to include injuries, interruption in education etc.*

**Response 9**

The statement has been rephrased to ensure clarity, and another reference added.

10. *"However, although these results are important, they are fragmented and limited in their collation of evidence, making it difficult to draw conclusions for policy and practice." It should be more explicitly explained why the prior reviews are insufficient and a new review is needed. This is unclear.*

**Response 10**

The paragraph is comprehensively improved.

11. "These reviews concluded that adolescents in vulnerable populations, especially those living in low-income areas, are disproportionately affected by climate change events." Only adolescents were found affected? What about younger children?
    And per next sentence, are the effects mostly mental health? One referenced article (#27) is from 2014, well before the reviews were conducted.

**Response 11**

These statements are revised to address the issue raised.

12. *" The health impacts on adolescents and children in low- and middle-income countries (LMICs), indigenous communities, and individuals with preexisting health conditions are also not addressed in the existing reviews." What about ref #8?*
    **Response 12**

The statements have been rephrased to address the issues raised. We have removed ref #8 from this statement.

I am unable to assess the methods due to lack of expertise. This should be assessed by a different reviewer.

**REVIEWER #2: Comments and Responses**

Thank you for asking me to review this protocol. I would like to congratulate the authors on this interesting and important research topic. I have some suggestions for consideration.

1. *As you have proposed an umbrella review (review of reviews), shouldn’t that be a systematic review? What is your justification for your scoping review?*

**Response 1**

A scoping review of reviews is used because the aim is to map the breadth and scope of the existing review literature on the topic. It does not aim to synthesize conclusions or produce a definitive answer about effectiveness; it is exploratory rather than evaluative.

2. *There are a range of terms used when it comes to climate change such as planetary health, environmental sustainability, environmental change etc. It would be good to flag there and talk about how these align or vary across the overarching concept of climate change.*

**Response 2**
The suggested range of terms are included to avoid missing relevant reviews that use different but related terminology.

3. *Why particular focus on adolescents in the research question? Wouldn’t the broad term of “children” capture all (infants, toddlers, teenagers etc)?*

**Response 3**

The terms children and adolescents are used together throughout this work because their age ranges overlap across major international frameworks; the WHO and UNICEF define children as those below 18, while adolescents are defined as those aged 10 to 19. Since neither term alone adequately captures both groups in the literature, the two are used to ensure inclusive and accurate representation

4. *What is JSTOR?*

**Response 4**

Journal Storage (JSTOR) is a digital library and online database that provides access to thousands of academic journals, books, and primary sources across a wide range of disciplines, including the humanities, social sciences, and natural sciences. It is widely used by researchers, students, and educators for accessing peer-reviewed scholarly content.

5. *What about other databases such as CINAHL, Embase, Cochrane Library etc?*

**Response 5**

Six of the major databases were used; however, the Cochrane Library has been added to the list of databases to be searched.

6. *Why the date limit from January 2020? Who says literature published since then is contemporary? What about December 2019?*

**Response 6**

The search period is designed to capture reviews between January 2000 and December 2025. That is a period of 25 years.

7. *Will there be any other limits imposed such as language?*

**Response 7**

Yes, only reviews conducted in the English language will be considered.

8. *Why not search organisational websites, forward and backward citation etc?*

**Response 8**

The primary purpose of searching organizational websites (e.g., WHO, UNICEF, government ministries) is to retrieve reports, guidelines, and policy documents, content produced outside academic publishing pipelines and not peer-reviewed. This directly conflicts with the study's exclusion of grey literature.

9. *Are you not using any keywords or subject headings for reviews?*

**Response 9**

Keywords or subject headings for reviews have been included, and the search strategy has been improved.

10. *What is your understanding of grey literature as Google and Google Scholar are grey literature and you are planning on excluding grey literature?*

**Response 10**

Grey literature is any research work or information that has not undergone formal peer review or passed through traditional academic publishing channels. This study is a review of reviews; therefore, any research other than reviews is excluded. Similarly, reports, conference proceedings, and editorials are excluded.

11. *How will you account for the same publications appearing in multiple reviews? Are you planning on undertaking a CCA (the Corrected Covered Area (CCA) is a metric used in umbrella reviews to quantify the degree of overlap (shared primary studies) among multiple systematic reviews)?*

**Response 11**

The Corrected Covered Area (CCA) would not be used because this review does not focus on the individual studies included in the various reviews. It is aimed at mapping the breadth and scope of existing reviews to identify gaps, inconsistencies, and future research priorities.

We thank the reviewers.

Rilwan Yahaya

Corresponding Author
