# Supplementary material for: Health impacts of climate change on children and adolescents: A protocol for review of reviews
Source: PLoS One. 2026 Jul 2;21(7):e0352978. doi: 10.1371/journal.pone.0352978 (PMC13327113; doi:10.1371/journal.pone.0352978)
Supplement: Peer review history

# Author comment: Author response to Decision Letter 0

**DOI:** 10.1371/journal.pone.0352978.r001

**Transfer Alert**

This paper was transferred from another journal. As a result, its full editorial history (including decision letters, peer reviews and author responses) may not be present.

16 Jan 2026

# Peer review: Decision Letter 0

**DOI:** 10.1371/journal.pone.0352978.r002

**Authors:** LS Katrina Li

6 May 2026

PONE-D-25-69159

Health Impacts of Climate Change on Children and Adolescents: A Protocol for Review of Reviews

PLOS One

Dear Dr. YAHAYA,

Thank you for submitting your manuscript to PLOS ONE. After careful consideration, we feel that it has merit but does not fully meet PLOS ONE’s publication criteria as it currently stands. Therefore, we invite you to submit a revised version of the manuscript that addresses the points raised during the review process.

Please clearly address the points and suggestions raised by the two peer reviewers to improve clarity of this protocol and transparency of the proposed methodology of this umbrella review.

Additionally, please clarify if forward citation searching (i.e. search to see if the included studies are cited by more recent publications that may meet your review's eligibility criteria) will be conducted.

Please submit your revised manuscript by Jun 20 2026 11:59PM. If you will need more time than this to complete your revisions, please reply to this message or contact the journal office at plosone@plos.org. When you're ready to submit your revision, log on to https://www.editorialmanager.com/pone/ and select the 'Submissions Needing Revision' folder to locate your manuscript file.

Please include the following items when submitting your revised manuscript:

- A letter that responds to each point raised by the academic editor and reviewer(s). You should upload this letter as a separate file labeled 'Response to Reviewers'.
- A marked-up copy of your manuscript that highlights changes made to the original version. You should upload this as a separate file labeled 'Revised Manuscript with Track Changes'.
- An unmarked version of your revised paper without tracked changes. You should upload this as a separate file labeled 'Manuscript'.

If you would like to make changes to your financial disclosure, please include your updated statement in your cover letter. Guidelines for resubmitting your figure files are available below the reviewer comments at the end of this letter.

If applicable, we recommend that you deposit your laboratory protocols in protocols.io to enhance the reproducibility of your results. Protocols.io assigns your protocol its own identifier (DOI) so that it can be cited independently in the future. For instructions see: https://journals.plos.org/plosone/s/submission-guidelines#loc-laboratory-protocols. Additionally, PLOS ONE offers an option for publishing peer-reviewed Lab Protocol articles, which describe protocols hosted on protocols.io. Read more information on sharing protocols at https://plos.org/protocols?utm_medium=editorial-email&utm_source=authorletters&utm_campaign=protocols.

As the corresponding author, your ORCID iD is verified in the submission system and will appear in the published article. PLOS supports the use of ORCID, and we encourage all coauthors to register for an ORCID iD and use it as well. Please encourage your coauthors to verify their ORCID iD within the submission system before final acceptance, as unverified ORCID iDs will not appear in the published article. *Only*  the individual author can complete the verification step; PLOS staff *cannot*  verify ORCID iDs on behalf of authors.

We look forward to receiving your revised manuscript.

Kind regards,

LS Katrina Li

Academic Editor

PLOS One

Journal Requirements:

When submitting your revision, we need you to address these additional requirements.

1. Please ensure that your manuscript meets PLOS ONE's style requirements, including those for file naming. The PLOS ONE style templates can be found at

https://journals.plos.org/plosone/s/file?id=wjVg/PLOSOne_formatting_sample_main_body.pdf and

https://journals.plos.org/plosone/s/file?id=ba62/PLOSOne_formatting_sample_title_authors_affiliations.pdf

2. When completing the data availability statement of the submission form, you indicated that you will make your data available on acceptance. We strongly recommend all authors decide on a data sharing plan before acceptance, as the process can be lengthy and hold up publication timelines. Please note that, though access restrictions are acceptable now, your entire data will need to be made freely accessible if your manuscript is accepted for publication. This policy applies to all data except where public deposition would breach compliance with the protocol approved by your research ethics board. If you are unable to adhere to our open data policy, please kindly revise your statement to explain your reasoning and we will seek the editor's input on an exemption. Please be assured that, once you have provided your new statement, the assessment of your exemption will not hold up the peer review process.

3.  Please include captions for your Supporting Information files at the end of your manuscript, and update any in-text citations to match accordingly. Please see our Supporting Information guidelines for more information: http://journals.plos.org/plosone/s/supporting-information.

4. If the reviewer comments include a recommendation to cite specific previously published works, please review and evaluate these publications to determine whether they are relevant and should be cited. There is no requirement to cite these works unless the editor has indicated otherwise.

[Note: HTML markup is below. Please do not edit.]

Reviewers' comments:

Reviewer's Responses to Questions

**Comments to the Author**

1. Does the manuscript provide a valid rationale for the proposed study, with clearly identified and justified research questions?

The research question outlined is expected to address a valid academic problem or topic and contribute to the base of knowledge in the field.

Reviewer #1: Partly

Reviewer #2: Yes

**********

2. Is the protocol technically sound and planned in a manner that will lead to a meaningful outcome and allow testing the stated hypotheses?

The manuscript should describe the methods in sufficient detail to prevent undisclosed flexibility in the experimental procedure or analysis pipeline, including sufficient outcome-neutral conditions (e.g. necessary controls, absence of floor or ceiling effects) to test the proposed hypotheses and a statistical power analysis where applicable. As there may be aspects of the methodology and analysis which can only be refined once the work is undertaken, authors should outline potential assumptions and explicitly describe what aspects of the proposed analyses, if any, are exploratory.

Reviewer #1: Partly

Reviewer #2: Partly

**********

3. Is the methodology feasible and described in sufficient detail to allow the work to be replicable?

Descriptions of methods and materials in the protocol should be reported in sufficient detail for another researcher to reproduce all experiments and analyses. The protocol should describe the appropriate controls, sample size calculations, and replication needed to ensure that the data are robust and reproducible.

Reviewer #1: Yes

Reviewer #2: Yes

**********

4. Have the authors described where all data underlying the findings will be made available when the study is complete?

The PLOS Data policy requires authors to make all data underlying the findings described in their manuscript fully available without restriction, with rare exception, at the time of publication. The data should be provided as part of the manuscript or its supporting information, or deposited to a public repository. For example, in addition to summary statistics, the data points behind means, medians and variance measures should be available. If there are restrictions on publicly sharing data—e.g. participant privacy or use of data from a third party—those must be specified.

Reviewer #1: Yes

Reviewer #2: Yes

**********

5. Is the manuscript presented in an intelligible fashion and written in standard English?

PLOS ONE does not copyedit accepted manuscripts, so the language in submitted articles must be clear, correct, and unambiguous. Any typographical or grammatical errors should be corrected at revision, so please note any specific errors here.

Reviewer #1: No

Reviewer #2: Yes

**********

6. Review Comments to the Author

Please use the space provided to explain your answers to the questions above and, if applicable, provide comments about issues authors must address before this protocol can be accepted for publication. You may also include additional comments for the author, including concerns about research or publication ethics.

You may also provide optional suggestions and comments to authors that they might find helpful in planning their study.

(Please upload your review as an attachment if it exceeds 20,000 characters)

Reviewer #1: This will make a valuable contribution to the understanding of climate change and children's health. I am unclear if publication of the protocol is necessary or valuable prior to the review being completed.

The language is good but needs substantial revisions prior to publication. I am unable to name all the recommended changes due to number. The abstract and pages 8-10 need to be checked closely for grammar and fluency. I have listed below some specific comments, however these are not exhaustive.

Abstract-"Climate change events such as extreme heat, rising sea levels, floods, food insecurity, and others significantly affect local, regional, and global living conditions." These are not all "events"

"Therefore, this review aims to re-map the existing reviews of the impact of climate change on the health and well-being of children and adolescents." Have the reviews already been mapped? If not then this is a first "mapping" of all reviews rather than "re-map".

1. "Climate change refers to a gradual atmospheric shift"- I have not heard this description before. It may be accurate but I'm not certain.

References 5 and 6 likely should be national/international expert reports such as from IPCC/US National Climate Assessment rather than articles focused on single issue (agriculture) or by single author.

2. "Evidence suggests that the climate crisis is largely anthropogenic"- this should be more definitively stated. Per IPCC AR6 "Human activities, principally through emissions of greenhouse gases, have unequivocally

caused global warming"

3. "The rate and severity of life-threatening weather events..." the reference is only re extreme rainfall and drought, not the other listed weather events. Each of these events is different, and not all are increasing in both frequency and severity. References should support the statement for each listed event.

4. "the 2023 wildfires in Hawaii and the floods in Vermont were intense and frequent"-more frequent does not make sense here. It would be better to reference the article referenced by the book for this statement rather than the whole book.

5. Please confirm the accuracy of this statement. 3 billion is a high percentage of the world population. "Accordingly, heatwaves will displace up to three billion people by the end of this century [14]."

6. Page 9:"For example, the recent rise in malaria, malnutrition, diarrhea, mental health challenges, and

the risk of under-five mortality, as well as increases in wasting and stunting, are associated with climate change [21,23,24]." Are all of these increasing? I do not see that these references support the broad statement.

7. Page 9 is confusing for a variety of reasons.

- "implications for higher morbidity and mortality from various climate-related diseases"; "diseases" is not the correct term here, could be "conditions" to include injuries, interruption in education etc.

-"However, although these results are important, they are fragmented and limited in their collation of evidence, making it difficult to draw conclusions for policy and practice." It should be more explicitly explained why the prior reviews are insufficient and a new review is needed. This is unclear.

-"These reviews concluded that adolescents in vulnerable populations, especially those living in low-income areas, are disproportionately affected by climate change events." Only adolescents were found affected? What about younger children?

And per next sentence, are the effects mostly mental health? One referenced article (#27) is from 2014, well before the reviews were conducted.

-" The health impacts on adolescents and children in low- and middle-income countries (LMICs), indigenous communities, and individuals with preexisting health conditions are also not addressed in the existing reviews." What about ref #8?

I am unable to assess the methods due to lack of expertise. This should be assessed by a different reviewer.

Reviewer #2: Thank you for asking me to review this protocol. I would like to congratulate the authors on this interesting and important research topic. I have some suggestions for consideration.

• As you are proposed an umbrella review (review of reviews), shouldn’t that be a systematic review? What is your justification for your scoping review?

• There are a range of terms used when it comes to climate change such as planetary health, environmental sustainability, environmental change etc. It would be good to flag there and talk about how these align or vary across the overarching concept of climate change.

• Why particular focus on adolescents in the research question? Wouldn’t the broad term of “children” capture all (infants, toddlers, teenagers etc)?

• What is JSTOR?

• What about other databases such as CINAHL, Embase, Cochrane Library etc?

• Why the date limit from January 2020? Who says literature published since then is contemporary? What about December 2019?

• Will there be any other limits imposed such as language?

• Why not search organisational websites, forward and backward citation etc?

• Are you not using any keywords or subject headings for reviews?

• What is your understanding of grey literature as Google and Google Scholar are grey literature and you are planning on excluding grey literature?

• How will you account for the same publications appearing in multiple reviews? Are you planning on undertaking a CCA (the Corrected Covered Area (CCA) is a metric used in umbrella reviews to quantify the degree of overlap (shared primary studies) among multiple systematic reviews)?

**********

7. PLOS authors have the option to publish the peer review history of their article (what does this mean?). If published, this will include your full peer review and any attached files.

If you choose “no”, your identity will remain anonymous but your review may still be made public.

**Do you want your identity to be public for this peer review?**  For information about this choice, including consent withdrawal, please see our Privacy Policy.

Reviewer #1: No

Reviewer #2: No

**********

[NOTE: If reviewer comments were submitted as an attachment file, they will be attached to this email and accessible via the submission site. Please log into your account, locate the manuscript record, and check for the action link "View Attachments". If this link does not appear, there are no attachment files.]

To ensure your figures meet our technical requirements, please review our figure guidelines: https://journals.plos.org/plosone/s/figures

You may also use PLOS’s free figure tool, NAAS, to help you prepare publication quality figures: https://journals.plos.org/plosone/s/figures#loc-tools-for-figure-preparation.

NAAS will assess whether your figures meet our technical requirements by comparing each figure against our figure specifications.

# Author comment: Author response to Decision Letter 1

**DOI:** 10.1371/journal.pone.0352978.r003

15 Jun 2026

Response to Editor and Reviewers

Manuscript title: Health Impacts of Climate Change on Children and Adolescents: A Protocol for Review of Reviews

Journal: PLOS ONE

Manuscript ID: PONE-D-25-69159

Date: 30th May 2026

Dear Katrina Li,

Thank you for the opportunity to revise and resubmit our manuscript. We are grateful to the Editor and Reviewers for their thorough and constructive comments. The feedback has significantly strengthened the manuscript's rigor, clarity, and comprehensiveness.

Below, we provide a point-by-point response to each comment. All changes to the manuscript are indicated in the revised version. For ease of reference, each comment is reproduced in full, followed by our response and description of the corresponding revision.

REVIEWER #1: Comments and Responses

This will make a valuable contribution to the understanding of climate change and children's health. I am unclear if publication of the protocol is necessary or valuable prior to the review being completed.

1. The language is good but needs substantial revisions prior to publication. I am unable to name all the recommended changes due to number. The abstract and pages 8-10 need to be checked closely for grammar and fluency. I have listed below some specific comments, however these are not exhaustive.

Response 1

Issues with grammar and fluency in the Abstract and on pages 8-10 have been addressed.

2. Abstract-"Climate change events such as extreme heat, rising sea levels, floods, food insecurity, and others significantly affect local, regional, and global living conditions." These are not all "events"

Response 2

We have addressed this comment.

3. "Therefore, this review aims to re-map the existing reviews of the impact of climate change on the health and well-being of children and adolescents." Have the reviews already been mapped? If not then this is a first "mapping" of all reviews rather than "re-map".

"Climate change refers to a gradual atmospheric shift"- I have not heard this description before. It may be accurate but I'm not certain.

Response 3

We addressed the sentence on “remap” to reflect our intention of synthesizing existing revies.

Yes, our description of climate change is correct, but we provided further explanation for clarity.

4. References 5 and 6 likely should be national/international expert reports such as from IPCC/US National Climate Assessment rather than articles focused on single issue (agriculture) or by single author.

Response 4

References 5 and 6 have been improved to include expert reports.

5. "Evidence suggests that the climate crisis is largely anthropogenic"- this should be more definitively stated. Per IPCC AR6 "Human activities, principally through emissions of greenhouse gases, have unequivocally

caused global warming"

Response 5

This statement has been improved to address the concern raised.

6. "The rate and severity of life-threatening weather events..." the reference is only re extreme rainfall and drought, not the other listed weather events. Each of these events is different, and not all are increasing in both frequency and severity. References should support the statement for each listed event.

Response 6

More references have been added to support this statement.

7. "the 2023 wildfires in Hawaii and the floods in Vermont were intense and frequent"-more frequent does not make sense here. It would be better to reference the article referenced by the book for this statement rather than the whole book.

Response 7

The statement has been improved and an additional reference added.

8. Please confirm the accuracy of this statement. 3 billion is a high percentage of the world population. "Accordingly, heatwaves will displace up to three billion people by the end of this century [14]."

Response 8

The statements have been rephrased to clarify their accuracy.

9. Page 9:"For example, the recent rise in malaria, malnutrition, diarrhea, mental health challenges, and the risk of under-five mortality, as well as increases in wasting and stunting, are associated with climate change [21,23,24]." Are all of these increasing? I do not see that these references support the broad statement.

7. Page 9 is confusing for a variety of reasons.

- "implications for higher morbidity and mortality from various climate-related diseases"; "diseases" is not the correct term here, could be "conditions" to include injuries, interruption in education etc.

Response 9

The statement has been rephrased to ensure clarity, and another reference added.

10. "However, although these results are important, they are fragmented and limited in their collation of evidence, making it difficult to draw conclusions for policy and practice." It should be more explicitly explained why the prior reviews are insufficient and a new review is needed. This is unclear.

Response 10

The paragraph is comprehensively improved.

11. "These reviews concluded that adolescents in vulnerable populations, especially those living in low-income areas, are disproportionately affected by climate change events." Only adolescents were found affected? What about younger children?

And per next sentence, are the effects mostly mental health? One referenced article (#27) is from 2014, well before the reviews were conducted.

Response 11

These statements are revised to address the issue raised.

12. " The health impacts on adolescents and children in low- and middle-income countries (LMICs), indigenous communities, and individuals with preexisting health conditions are also not addressed in the existing reviews." What about ref #8?

Response 12

The statements have been rephrased to address the issues raised. We have removed ref #8 from this statement.

I am unable to assess the methods due to lack of expertise. This should be assessed by a different reviewer.

REVIEWER #2: Comments and Responses

Thank you for asking me to review this protocol. I would like to congratulate the authors on this interesting and important research topic. I have some suggestions for consideration.

1. As you have proposed an umbrella review (review of reviews), shouldn’t that be a systematic review? What is your justification for your scoping review?

Response 1

A scoping review of reviews is used because the aim is to map the breadth and scope of the existing review literature on the topic. It does not aim to synthesize conclusions or produce a definitive answer about effectiveness; it is exploratory rather than evaluative.

2. There are a range of terms used when it comes to climate change such as planetary health, environmental sustainability, environmental change etc. It would be good to flag there and talk about how these align or vary across the overarching concept of climate change.

Response 2

The suggested range of terms are included to avoid missing relevant reviews that use different but related terminology.

3. Why particular focus on adolescents in the research question? Wouldn’t the broad term of “children” capture all (infants, toddlers, teenagers etc)?

Response 3

The terms children and adolescents are used together throughout this work because their age ranges overlap across major international frameworks; the WHO and UNICEF define children as those below 18, while adolescents are defined as those aged 10 to 19. Since neither term alone adequately captures both groups in the literature, the two are used to ensure inclusive and accurate representation

4. What is JSTOR?

Response 4

Journal Storage (JSTOR) is a digital library and online database that provides access to thousands of academic journals, books, and primary sources across a wide range of disciplines, including the humanities, social sciences, and natural sciences. It is widely used by researchers, students, and educators for accessing peer-reviewed scholarly content.

5. What about other databases such as CINAHL, Embase, Cochrane Library etc?

Response 5

Six of the major databases were used; however, the Cochrane Library has been added to the list of databases to be searched.

6. Why the date limit from January 2020? Who says literature published since then is contemporary? What about December 2019?

Response 6

The search period is designed to capture reviews between January 2000 and December 2025. That is a period of 25 years.

7. Will there be any other limits imposed such as language?

Response 7

Yes, only reviews conducted in the English language will be considered.

8. Why not search organisational websites, forward and backward citation etc?

Response 8

The primary purpose of searching organizational websites (e.g., WHO, UNICEF, government ministries) is to retrieve reports, guidelines, and policy documents, content produced outside academic publishing pipelines and not peer-reviewed. This directly conflicts with the study's exclusion of grey literature.

9. Are you not using any keywords or subject headings for reviews?

Response 9

Keywords or subject headings for reviews have been included, and the search strategy has been improved.

10. What is your understanding of grey literature as Google and Google Scholar are grey literature and you are planning on excluding grey literature?

Response 10

Grey literature is any research work or information that has not undergone formal peer review or passed through traditional academic publishing channels. This study is a review of reviews; therefore, any research other than reviews is excluded. Similarly, reports, conference proceedings, and editorials are excluded.

11. How will you account for the same publications appearing in multiple reviews? Are you planning on undertaking a CCA (the Corrected Covered Area (CCA) is a metric used in umbrella reviews to quantify the degree of overlap (shared primary studies) among multiple systematic reviews)?

Response 11

The Corrected Covered Area (CCA) would not be used because this review does not focus on the individual studies included in the various reviews. It is aimed at mapping the breadth and scope of existing reviews to identify gaps, inconsistencies, and future research priorities.

We thank the reviewers.

Rilwan Yahaya

Corresponding Author

Attachment Submitted filename: Response to Reviewers.docx

# Editor report: Decision Letter 1

**DOI:** 10.1371/journal.pone.0352978.r004

**Authors:** LS Katrina Li, LS Katrina Li

18 Jun 2026

Health Impacts of Climate Change on Children and Adolescents: A Protocol for Review of Reviews

PONE-D-25-69159R1

Dear Dr. YAHAYA,

We’re pleased to inform you that your manuscript has been judged scientifically suitable for publication and will be formally accepted for publication once it meets all outstanding technical requirements.

Within one week, you’ll receive an e-mail detailing the required amendments. When these have been addressed, you’ll receive a formal acceptance letter and your manuscript will be scheduled for publication.

An invoice will be generated when your article is formally accepted. Please note, if your institution has a publishing partnership with PLOS and your article meets the relevant criteria, all or part of your publication costs will be covered. Please make sure your user information is up-to-date by logging into Editorial Manager at Editorial Manager® and clicking the ‘Update My Information' link at the top of the page. For questions related to billing, please contact billing support.

If your institution or institutions have a press office, please notify them about your upcoming paper to help maximize its impact. If they’ll be preparing press materials, please inform our press team as soon as possible -- no later than 48 hours after receiving the formal acceptance. Your manuscript will remain under strict press embargo until 2 pm Eastern Time on the date of publication. For more information, please contact onepress@plos.org.

Kind regards,

LS Katrina Li

Academic Editor

PLOS One

Additional Editor Comments (optional):

Reviewers' comments:

# Editor report: Acceptance letter

**DOI:** 10.1371/journal.pone.0352978.r005

**Authors:** LS Katrina Li, LS Katrina Li

PONE-D-25-69159R1

PLOS One

Dear Dr. YAHAYA,

I'm pleased to inform you that your manuscript has been deemed suitable for publication in PLOS One. Congratulations! Your manuscript is now being handed over to our production team.

At this stage, our production department will prepare your paper for publication. This includes ensuring the following:

* All references, tables, and figures are properly cited

* All relevant supporting information is included in the manuscript submission,

* There are no issues that prevent the paper from being properly typeset

You will receive further instructions from the production team, including instructions on how to review your proof when it is ready. Please keep in mind that we are working through a large volume of accepted articles, so please give us a few days to review your paper and let you know the next and final steps.

Lastly, if your institution or institutions have a press office, please let them know about your upcoming paper now to help maximize its impact. If they'll be preparing press materials, please inform our press team within the next 48 hours. Your manuscript will remain under strict press embargo until 2 pm Eastern Time on the date of publication. For more information, please contact onepress@plos.org.

You will receive an invoice from PLOS for your publication fee after your manuscript has reached the completed accept phase. If you receive an email requesting payment before acceptance or for any other service, this may be a phishing scheme. Learn how to identify phishing emails and protect your accounts at https://explore.plos.org/phishing.

If we can help with anything else, please email us at customercare@plos.org.

Thank you for submitting your work to PLOS ONE and supporting open access.

Kind regards,

PLOS ONE Editorial Office Staff

on behalf of

Dr. LS Katrina Li

Academic Editor

PLOS One
